# Pattern of Crimean-Congo hemorrhagic fever related high risk behaviors among Iranian butchers and its relation to perceived self-efficacy

**DOI:** 10.1186/s12889-021-10333-7

**Published:** 2021-01-30

**Authors:** Davoud Adham, Malek Abazari, Eslam Moradi-Asl, Abbas Abbasi-Ghahramanloo

**Affiliations:** 1 Department of Public Health, School of Public Health, Ardabil University of Medical Sciences, Ardabil, Iran; 2 Arthropod Borne Diseases Research Center, Ardabil University of Medical Sciences, Ardabil, Iran

**Keywords:** Crimean-Congo hemorrhagic fever, Butchers, Self-efficacy, Iran

## Abstract

**Background:**

Crimean-Congo hemorrhagic fever (CCHF) is highly fatal to humans and an acute viral disease. The CCHF disease has been reported in occupations such as butchers, slaughterhouse butchers and workers. The aim of this study was to investigate the pattern of CCHF related high risk behaviors among butchers and determine the effects of perceived self-efficacy of the participants on their membership in latent classes.

**Methods:**

The participants of this cross-sectional study were recruited from all the meat distribution centers in Ardabil Province in 2019–2020. The LCA approach was used to observe the CCFH related high risk behaviors patterns.

**Results:**

The statistical significance level was assigned at *P*-value < 0.05 in all the analyses. Three latent classes were identified; namely, 1) low risk (16.1%), 2) high risk (53.6%), and 3) very high risk (30.2%). After adjusting for other possible confounders higher score of perceived self-efficacy significantly decrease the odds of membership in high risk class (OR = 0.74) and very high risk class (OR = 0.62) compared to the low risk class. Also, age (OR = 1.07) and experience (OR = 0.91) associate with very high risk class.

**Conclusions:**

This study revealed the co-occurrence of CCHF related high risk behaviors in the majority of workers in the livestock and meat industry. It is necessary to provide butchers and slaughterhouse workers with general education, force them to use protective equipment and investigate the rate of tick bites in risky occupations.

**Supplementary Information:**

The online version contains supplementary material available at 10.1186/s12889-021-10333-7.

## Background

Crimean-Congo hemorrhagic fever (CCHF) is an acute viral disease. This disease is highly fatal to humans and has been reported from over 50 European, Asian or African countries around the world [1, 2]. CCHF can also inflict other domestic and wild animals with no clinical symptoms, though. The disease is caused by the CCHF virus that is an Orthonairovirus genus belonging to the order Bunyavirales in the family Nairoviridae [3]. Humans are usually infected with Crimean-Congo hemorrhagic fever virus (CCHFV) via having tick biting,direct contact with the blood, mucus and tissue of infected livestock or contact with the bodily fluids or personal belongings of another person suffering from the disease [3, 4].

The vectors of CCHF are usually hard ticks with the genus *Hyalomma marginatum* as the main vectors [5, 6]. One way to avoid and control the disease is to use insecticides and safe repellents [7, 8]. The majority of the infected patients are farmers, veterinarians, hospital staff and slaughterhouse butchers and workers [9, 10]. If a person has been in direct, unprotected contact with the blood, fluids or tissue of an infected animal or in direct contact with the bodily fluids of an infected person, or if they have stayed in rural areas, they can be regarded as a suspicious case [11].

Unpublished reports by Ardabil Province health centers show that a CCHF outbreak occurred in Ardabil Province with 50 suspicious cases and 10 positive ones leading to one death in total. Butchers are amongst one of the most susceptible groups of people who are exposed to the disease. Thus, it is of crucial importance to investigate and identify dangerous behavioral patterns regarding CCHF in this group of individuals.

Latent class analysis (LCA) is a method to identify subclasses or latent classes amongst the participants of a study. This method is person-centered and uses observable and classifiable variables to identify subclasses [12]. The present study used a LCA to investigate potential subgroups of butchers based on their responses to a series of questions about CCFH related high risk behaviors. Since to the best of the researchers’ knowledge, no similar study has been conducted in this regard, this study aimed to investigate the pattern of CCHF related high risk behaviors among butchers and determine the effects of perceived self-efficacy of the participants on their membership in latent classes in Ardabil Province.

## Methods

### Study area

The participants of this cross-sectional study were recruited from all the meat distribution centers in Ardabil Province. This province is located in the northwest of Iran. A multistage sampling method was employed to select these centers. In the first stage, each city in the province was regarded as one stratum. In the second stage, each city was divided into four strata, and then a sufficient number of participants were selected from each stratum based on convenience sampling method. A standard questionnaire consisting of different sections was used to gather the required data [4]. In this study, two sections of the standard questionnaire were used: one section focusing on dangerous behaviors regarding CCHF and the other section focusing on self-efficacy. The Cronbach’s Alpha coefficients of high risk behaviors and self-efficacy sections were 0.67 and 0.77, respectively. Used sections of this questionnaire attached as an additional file to this paper.

### Statistical analysis

The LCA model was used to observe the CCFH related high risk behaviors patterns. In this statistical model, several observed variables are used aggregately so that the overall pattern of the latent variable is identified. In this model, latent and observed variables are all categorical. To select the best model, some statistical indices are utilized such as AIC, BIC and G2. The lower these indices are, the better the model will be; hence, a model with the lowest AIC, BIC and G2 can be regarded as the best model.

To describe the characteristics of each latent class, probabilities beyond 0.5 were emphasized. The latent classes were also labeled based on probabilities beyond 0.5. In this study, seven observed variables were used. The labeled observed variables included the following: 1) having a history of contact with infected animals’ carcass, blood or red meat, 2) not using protective gloves while dressing animals or having contact with animals’ blood or carcass, 3) not using protective masks while dressing animals or having contact with animals’ blood or carcass, 4) not using protective goggles while dressing animals or having contact with animals’ blood or carcass, 5) not using work clothes and boots while dressing animals or having contact with animals’ blood or carcass, 6) holding the knife in the mouth while dressing animals or having contact with animals’ blood or carcass and 7) having a history of physical contact with ticks.

After the finalization of the model, the authors entered the following covariates into the model: age, experience, residency, education, marital status and perceived self-efficacy. The SPSS 16 software was used to report the frequencies of observed variables, and the SAS 9.2 software was used to conduct LCA analysis via PROC LCA. The statistical significance level was assigned at *P*-value < 0.05 in all the analyses.

## Results

A total of 420 slaughterhouse butchers participated in this study. The mean age of the participants was 40.44 ± 10.23 SD. Moreover, the mean work experience of the participants was 14.52. The results revealed that 49% of the butchers lived in rural areas, 17.1% were not married and 13.8 were illiterate. Table 1 show the prevalence of each high risk behavior.

**Table 1:** Demographic characteristics and prevalence of CCHF related high risk behaviors in a sample of Iranian butchers

Items	Total (*n* = 420)
	N (%)
**Sex**	
Male	400 (95.2)
Female	16 (3.8)
**Region**	
Rural	206 (49.0)
Urban	214 (51.0)
**Education**	
≥ 12 years	364 (86.7)
< 12 years	56 (13.3)
**Marital status**	
Single	72 (17.1)
Married	348 (82.9)
**Having history contact with carcasses, blood or raw red meat**	352 (83.8)
**Skipping of gloves wearing**	220 (52.4)
**Skipping of mask wearing**	336 (80.0)
**Skipping of glasses wearing**	348 (82.9)
**Skipping of using work clothes and boots**	120 (28.6)
**Knife carrying with mouth**	32 (7.6)
**Having history of physical contact with ticks**	170 (40.5)

Given the seven observed variables, there could exist 128 response patterns. The fitness of the LCA model was investigated by seven variables for one-class to six-class models, and the relevant indices of each model are presented in Table 2. According to the fitness indices and interpretability of the results, the three-class model was preferred in this study. Table 3 shows the LCA model’s output for the three classes. This table consists of two sections. The first section shows the prevalence of each of the latent classes. As observed in Table 3, 16.1% of the participants were located in the first class, 53.6% in the second class and 30.2% in the third class. The second section of Table 3 depicts the probability of each of the indicator variables. To present a more exact description of each class, the detailed characteristics of each class need to be elaborated:

**Table 2:** Comparison of LCA Models With Different Latent Classes Based on Model Selection Statistics

Number of latent class	Number of parameters estimated	G^2^	df	AIC	BIC	Entropy	Maximum log-likelihood
1	7	656.85	120	670.85	699.07	1.00	− 1482.09
2	15	205.99	112	235.99	296.45	0.93	− 1256.65
**3**	**23**	**115.42**	**104**	**161.42**	**254.13**	**0.90**	**− 1211.37**
4	31	87.78	96	149.78	274.73	0.92	− 1197.55
5	39	61.44	88	139.44	296.63	0.87	− 1184.38
6	45	42.14	80	136.14	325.58	0.81	− 1174.73

*Note*. *LCA* latent class analysis, *AIC* Akaike information criterion, *BIC* Bayesian information criterion

**Table 3:** The three Latent Classes Model of CCHF related high risk behaviors in a sample of Iranian butchers

Items	Latent class		
	Low risk	High risk	Very high risk
**Latent class prevalence**	0.161	0.536	0.302
**Item-response probabilities**			
Having history contact with carcasses, blood or raw red meat	0.440	**0.889**	**1.00**
Skipping of gloves wearing	0.007	0.487	**0.900**
Skipping of mask wearing	0.052	**0.934**	**0.999**
Skipping of glasses wearing	0.119	**0.978**	**0.983**
Skipping of using work clothes and boots	0.031	0.002	**0.988**
Knife carrying with mouth	0.178	0.042	0.086
Having history of physical contact with ticks	0.072	**0.501**	0.428

Note. Item-response probabilities >.5 in bold to facilitate interpretation

### The first class (low-risk)

The probability of the occurrence of high risk behaviors to the participants in this class was low and below 50%. Nevertheless, it should be mentioned that the probability of having a history of contact with animals’ carcass, blood or red meat was 44% in this class, which is still a noticeable amount. Yet, the probability of not using protective gloves, masks and goggles as well as having a history of physical contact with ticks was the lowest amount possible across all classes.

### The second class (high-risk)

The probability of not using gloves, work clothes and boots as well as having contact with animals or holding the knife in the mouth while dressing animals was below 50% in this class. However, other high risk behaviors had a high probability in this class. It needs to be mentioned that not using work clothes and boots as well as holding the knife in the mouth while dressing animals in this class had the lowest probability across all classes.

### The third class (very high-risk)

The probability of holding the knife in the mouth was low in this class. Likewise, having a history of physical contact with ticks was below 50% in this class. The other observed variables, however, had a high probability amount so that the probability of a history of contact with animals’ carcass, blood or red meat was 100% in this class. Similarly, the probability of not using protective gloves, masks and goggles were 90%, about 100 and 98.3%, respectively, in this class.

Table 4 demonstrates the odds ratios of membership in each latent class. As can be seen, an increase in age raised the odds of being in the third class by 1.07. On the other hand, an increase in work experience could decrease the odds of being in the third class by 0.91. The results of the study revealed that by controlling the effect of other variables, the perceived self-efficacy score could decrease the odds of being in the second and third classes compared to the first class. Hence, the odds of being in latent class 2 and 3 in comparison to the class 1 was 0.74 and 0.62 respectively.

**Table 4:** Predictors of membership in latent classes of CCHF related high risk behaviors

Predictors	Low risk	High risk	Very high risk	***P***-value
	OR(95%CI)	OR(95%CI)	OR(95%CI)	
Age (year)	Reference	1.01(0.97–1.05)	1.07(1.02–1.13)	0.0112
Experience (year)	Reference	0.99(0.96–1.03)	0.91(0.86–0.95)	< 0.001
Residency (being rural citizenship)	Reference	1.26(0.70–2.27)	2.10(1.05–4.17)	0.0844
Education (being uneducated)	Reference	1.37(0.66–2.84)	1.32(0.58–3.00)	0.8357
Marital status (being single)	Reference	0.74(0.21–2.65)	0.42(0.09–1.97)	0.5132
Score of Perceived Self efficacy	Reference	0.74(0.65–0.83)	0.62(0.54–0.72)	< 0.001

## Discussion

The CCHF disease has been frequently reported in several occupations such as butchers, slaughterhouse butchers and workers, doctors, veterinarians, hospital staffs and laboratory employees [4, 13]. It has been observed that occupation related high risk behaviors can increase the risk of infection more than the personal awareness and performance of the individuals [14, 15]. Typical high risk behaviors exhibited by slaughterhouse butchers include eating raw liver, holding the knife in the mouth while dressing animals and not wearing appropriate work clothes and boots [16]. The results of this study revealed that having a history of contact with animals’ raw meat, blood and carcass without any appropriate protection is the most prevalent type of CCHF related high risk behavior among slaughterhouse butchers (88.8%). Also, not using protective goggles (82.9%) and masks (80%), which are amongst the most dangerous types of behavior for transmitting the CCHF disease, are the next two prevalent high risk behaviors. Using protective covers such as protective gloves, masks, goggles, work clothes and boots while slaughtering or dressing livestock or having contact with their meat products can prevent the transmission of the disease to humans via the skin or tissue. Such protective behaviors are also considered as some helpful preventive measures. These protective measures are important in butcheries and slaughterhouses and can even prove useful in preventing the transmission of the disease to veterinarians and cooks. Studies in Ilam and Mazandaran provinces in Iran have reported that 75.3 and 73.8% of respondents do not use protective covers while having contact with livestock or cleaning their platform [17, 18]. Likewise, research has revealed that 38% of CCHF positive cases in Khorasan Province and 5.9% of CCHF positive cases in Qom Province have mentioned that they have had contact with the blood or carcass of the livestock or have eaten their raw liver [5, 19]. Providing appropriate education to these individuals can help them change their behavioral patterns. As an example, a study in Turkey shows that the literacy level of the individuals directly affects the way they use protective covers [20].

The findings of this study demonstrated that 40.5% of the respondents confirm having physical contact with ticks. Ticks’ bite is the main and most effective way through which the CCHF disease is transmitted. One of the most dangerous behaviors exhibited by livestock farmers and slaughterhouse butchers around the world is removing ticks from the skin of livestock without wearing any protective gloves. For example, 34.3% of the individuals in Turkey and 14.7% of the individuals in Qom Province, Iran, have reported such a dangerous behavior [19].

The results of the LCA model showed that only 16.1% of the participants are in the low-risk class. However, about 54% of the participants are placed in the high-risk class and 30% in the very high-risk class. In other words, around 84% of the slaughterhouse butchers are in the high-risk and very high-risk classes. Given the co-occurrence of CCHF related high risk behaviors in these two groups, individuals belonging to these two groups are highly susceptible to being infected with the CCHF disease. Research shows that 79% of butchers are aware of the fact that they run the risk of contracting zoonotic diseases [2, 21]. Therefore, implementing appropriate, preventive interventions are crucial in this occupation. Accordingly, high self-efficacy can act as a preventive factor against CCHF related high risk behaviors [22, 23]. The results of the current study also showed that perceived self-efficacy can reduce the odds of being in the high-risk or very high-risk groups. Therefore, it is expected that appropriate interventions aiming at increasing self-efficacy can eventually increase the occurrence of protective behaviors such as using suitable gloves, goggles and other necessary protective measures in butchers.

## Conclusion

The high prevalence of the CCHF disease in livestock farms, butcheries and slaughterhouses proves the relationship between such occupations and hard ticks as the main vectors of the disease. Since there are no vaccines or medicines available to the public, and the results of this study revealed the co-occurrence of CCHF related high risk behaviors in the majority of workers in the livestock and meat industry; such behaviors can increase the incidence rate of the disease or even result in its epidemic. Thus, it is necessary to provide butchers and slaughterhouse workers with general education, force them to use protective equipment and investigate the rate of tick bites in risky occupations.

## Supplementary Information

**Additional file 1.** Questionnaire file.

## Data Availability

The data collection tools and datasets generated and/or analyzed during the current study are available from the corresponding author on reasonable request.

## Abbreviations

CCHF: Crimean-Congo hemorrhagic fever
LCA: Latent class analysis
AIC: Akaike Information Criteria
BIC: Bayesian Information Criteria
