# Supplementary material for: Pattern of Crimean-Congo hemorrhagic fever related high risk behaviors among Iranian butchers and its relation to perceived self-efficacy
Source: BMC Public Health. 2021 Jan 30;21:255. doi: 10.1186/s12889-021-10333-7 (PMC7847603; doi:10.1186/s12889-021-10333-7)
Supplement: Supplementary file 1 — Additional file 1. Questionnaire file. [file 12889_2021_10333_MOESM1_ESM.pdf]

Perceived self-efficacy	Completely agree	agree	disagree	completely disagree
I can easily wear glasses during slaughter.				
I can always use gloves to touch and slaughter the animal.				
I can use a proper mask at the time of slaughter.				
I can easily wear boots and work clothes before slaughter.				

High risk behaviors	yes	no
Did you have a history of contact with carcasses, blood or raw red meat?		
Do you always use gloves when slaughtering livestock or in contact with blood and carcasses?		
Do you always wear a mask when slaughtering animals or in contact with blood and carcasses?		
Do you always wear appropriate glasses when slaughtering livestock or in contact with blood and carcasses?		
Do you always use work clothes and boots when slaughtering livestock or in contact with blood and carcasses?		
Do you always carry a knife with your mouth when slaughtering animals or contacting blood and carcasses?		
Do you have a history of physical contact with ticks?		
